# Dentistry as a professional career: the views of London's secondary school pupils (2011-2017)

**DOI:** 10.1038/s41415-022-4044-x

**Published:** 2022-03-25

**Authors:** Victoria Niven, Lyndon B. Cabot, Sasha Scambler, Jennifer E. Gallagher

**Affiliations:** 4141598893001 Teacher in Dental Public Health, King´s College London, Faculty of Dentistry, Oral and Craniofacial Sciences, King´s College London, Bessemer Road, London, SE5 9RS, UK; 4141598893002 Associate Dean, Undergraduate Clinical Education and Honorary Consultant GSTT Trust Service Lead for Undergraduate Activity, Guy´s Hospital, Great Maze Pond, London, SE1 1UL, UK; 4141598893003 Reader in Medical Sociology and Academic Lead for Equality, Diversity and Inclusion, Faculty of Dentistry, Oral and Craniofacial Sciences, King´s College London, Central Office, Floor 18, Tower Wing, Guy´s Campus, London, UK; 4141598893004 Global Envoy King´s College London; Dean for International Affairs; Newland-Pedley Professor of Oral Health Strategy; Honorary Consultant in Dental Public Health; and Discipline Lead for Dental Public Health, Faculty of Dentistry, Oral and Craniofacial Sciences, King´s College London, Bessemer Road, London, SE5 9RS, UK

## Abstract

**Aim** To explore young people's perceptions of dentistry as a potential future career, including features which would attract or deter them from wanting to become dentists and the perceived influences on these views.

**Methods** Purposive sampling of London schools was undertaken. Exploration of academically-able, science-minded young people's (aged 14-18 years) perceptions of dentistry as a potential career was achieved through a series of focus groups conducted at various types of school in the Greater London region (13 focus groups and 91 students). A topic guide, informed by the literature and previous research, explored the perceived motivating and demotivating factors and associated influences, identified by these pupils, on studying dentistry at university. Data were analysed using framework methodology.

**Results** Multiple factors were identified by London secondary school pupils that would attract them to dentistry. Pull factors were: 1) science-based; 2) status and security - extrinsic rewards; 3) structure of service provision; 4) career opportunities; 5) social interactions; 6) personal skills and care - intrinsic rewards; and 7) being a vocational degree. Push factors away from the career included lack of diversity within the job and the 'negative image' of dentists, with medicine having greater social status and more varied career options. Individual and wider influences on pupils' perceptions included their personal experience with dentistry, social and community networks, the school environment, as well as system and societal level influencers.

**Conclusions** These findings suggest that a wide range of influences determine teenagers' perceptions of a dental career. Pupils in London schools report similar features of dentistry as being attractive as dental students, as well as its importance as a vocational degree, and although dentistry appears to lack status and profile when compared with medicine, it may be more acceptable in relation to its lifestyle. Individual sociodemographic characteristics and wider environmental factors may influence the relative importance of these features.

## Key points

- Provides a greater understanding of the features of a career in dentistry that attract/deter young people to/from this career early in their decision-making process.
- Explores how these perceptions may be influenced by individual characteristics.
- Describes the importance of the environmental context in influencing these perceptions.

## Background

Dentistry and medicine have been highly regarded professions in western society since UK higher education (HE) institutions first developed, but have been perceived as elitist^1^ and since the mid-nineteenth century, recognised as careers which improve social status.^2^ This perception of elitism can be countered with the argument that such healthcare professionals should reflect the populace they serve - the 'social, cultural and ethnic background of medical graduates should reflect broadly the diversity of patient population'.^3^^,^^4^ This increases our focus on who we are attracting to train as future doctors and dentists, not only to satisfy a HE widening participation agenda, but also to create a healthcare workforce which is socially accessible to both those who require and those who provide these services.^5^^,^^6^

Historically, by the end of the twentieth century, medicine and dentistry had a proven reputation as elite professions,^7^^,^^8^^,^^9^ with recent analysis of Universities and Colleges Admissions Service data confirming this continues in the twenty-first century.^10^^,^^11^ Research has revealed pupils who are Black, from non-selective state schools or from lower socioeconomic backgrounds were less likely to apply to study dentistry than their peers of white or Asian ethnicity, from selective schools and those from higher socioeconomic backgrounds.^12^^,^^13^Dentistry in recent years has diversified its student body, but Black students and those from lower socioeconomic groups remain underrepresented, both in applications and acceptances to dental school.^13^ It is crucial to explore why pupils from more disadvantaged backgrounds are not considering dentistry as a future career and listen to the 'student voices' from a spectrum of backgrounds.

Research exploring career decision making regarding dentistry has focused on two main fields - identifying perceived motivating features of the career and the influencers on this decision-making process. Studies investigating students' motivations to train to be dentists, both nationally and internationally, have been infrequent, with most research employing quantitative methodology and conducted on students already studying dentistry.^14^^,^^15^^,^^16^^,^^17^^,^^18^^,^^19^ Key factors identified were 'working with people', perceived ease of employment, being self-employed, working regular hours and financial opportunities as the most common motives to study dentistry, with having relatives or friends in the profession and 'lifelong ambition' as lesser factors.^14^^,^^20^ Although revealing, investigations identifying differences between students based on sociodemographic characteristics have been solely quantitative.^21^^,^^22^ The limited qualitative research in this area, although identifying push, pull and mediating factors involved in deciding upon a career in dentistry, has also only focused on those already studying dentistry or those recently qualified as dentists^23^^,^^24^^,^^25^ and has yet to identify demotivating features.

The importance of wider influences on choosing a career in dentistry (individual characteristics such as sex, ethnicity and maturity) have been found to be associated with differences in the students' views regarding the importance of motivating factors towards dentistry, with 'academic-scientific' factors being relatively more important to Asian students, 'family-friends' being more important to female students and those from minority ethnic groups and mature students being more concerned about 'careers-advising' factors.^6^^,^^24^ Modelling concepts of wider sociodemographic influences on dental career decision making, which has been applied in research exploring the motivations and influencers on medical student career decision making,^26^^,^^27^^,^^28^^,^^29^^,^^30^ has yet to be applied to dentistry.

Further research is required to explore the motivations of younger people earlier in their career decision-making process, from a spectrum of backgrounds, to inform stakeholders in reducing the underrepresentation of disadvantaged students and challenging the elitist reputation of dentistry.

## Aim

To explore the perceptions of young people from a spectrum of backgrounds on dentistry as a potential future career, including features which would attract or deter them from wanting to become dentists and the perceived influences on their career and degree choices.

## Method

Qualitative research was undertaken to explore the views of academically-able, science-minded young people (aged 14-18 years) and their perceptions of dentistry as a potential career, through a series of focus groups conducted in the school setting. Ethics committee approval was sought and obtained from King's College London Research Ethics Committee (No. 10/11-17 and No. 14/15-40). Purposive sampling of London schools (state, independent, grammar, further education college [FEC] and academy) was undertaken across the academic spectrum (high and low performance scores) through publicly available lists of schools by London local authority and published league tables. Schools were approached via head teachers and invited to participate in the study, with each school providing written informed consent and selecting the students to participate in focus groups. All students (and parents of students below 16 years of age) were provided with an information sheet and consent form. Written consent was obtained from the pupils, directly if they were aged 16 years and over, or if they were aged under 16 years, written parental consent was obtained, to participate in the study and for anonymous data to be published. A topic guide, informed by the literature and previous research,^26^^,^^28^ explored the perceived features that would attract/deter students to/from a career in dentistry and identified sociodemographic factors which students perceived would influence their choice of degree. The focus groups opened with an icebreaker to stimulate discussion (for example, each students' plans for the summer holidays). Students were informed at the outset that any questions about university would be dealt with once the focus group had ended. Focus group discussions were taped and transcribed for analysis. Data were analysed using framework methodology^31^ which has been successfully used in motivational research.^23^^,^^24^^,^^32^ Framework methodology provided a matrix-based system which allowed for systematic and visible stages to the data analysis process: familiarisation; identification of a provisional thematic framework; indexing; charting; and mapping and interpretation.^33^

## Results

### Participants

All of the participating schools provided GCSE and A-level education, with the FEC providing GCSE education for adults only. Of the A-level participants, 33 out of the 71 participants were hoping to study medicine, with 14 hoping to study dentistry. It is interesting to note that at the end of Year 12, six pupils were still undecided over their career choice and a further five were still choosing between dentistry and medicine. The majority of the GCSE pupils identified they wished to study medicine at university (n = 11), with four hoping to study dentistry. All of the focus groups composed prospective medical (m) and dental (d) pupils and this allowed for the exploration of the interplay in the career decision-making influences between the two groups, particularly in relation to the motivating and demotivating features of both careers.

Of the remaining academically-able informants, ten stated that they did not yet know what they wished to study at university, with 14 pupils stating they were hoping to study courses other than medicine or dentistry. These courses included: chemical engineering; biochemistry; chemistry; biology; physics; biomedical sciences; pharmacy; nursing; philosophy, politics and economics; mathematics; fashion; and volcanology.

Of the A-level participants, half (n = 37) had parents in higher managerial professions, with the pupils from the independent schools having parents exclusively from this socioeconomic group. However, the range of parental occupations of the remaining focus groups was broad. As the majority of Asian UK applicants to dentistry have been shown to be from India, this ethnic group was separated to identify pupils of Indian and other Asian groups (South Korean, Sri Lankan, Chinese, Pakistani and Bangladeshi).^10^ Of the participants in the FEC focus group, eight out of nine were of Bangladeshi ethnicity. By the nature of the school type stratification, further stratifications by sex, ethnicity and socioeconomic group were introduced - the participants within each school type were non-homogenous (see Table 1). Although within each focus group (with the exception of the independent schools) there was a range of socioeconomic groups, the pupils from the FEC were exclusively from medium and lower socioeconomic backgrounds.

**Table 1:** Characteristics of London secondary school pupils of different school types by socioeconomic and ethnic group

Academic stage	Socioeconomic group					Ethnicity				
School type	High	Medium	Low	Unknown	Unemployed	White	Asian - Indian	Asian - other	Black	Mixed ethnic group
**A-level pupils (A)**										
High state (↑s)	8	2	2	3	1	6	1	2	6	1
Low state (↓s)	5	1	1	0	0	0	1	4	2	0
High independent (↑i)	13	0	0	0	0	4	3	3	0	3
Low independent (↓i)	5	0	0	0	0	0	0	8	1	0
High grammar (↑g)	3	3	2	0	0	0	2	5	0	1
Low grammar (↓g)	4	1	0	1	1	0	5	2	0	0
Low FEC (↓f)	0	3	4	1	1	0	0	8	1	0
High academy (↑a)	2	1	2	1	0	5	0	1	0	0
**GCSE pupils (G)**										
State (s)	5	1				2	1	3		
Independent (i)	3	1		2		4	1			1
Grammar (g)	4	1	1	2			4	4		

Of the GCSE participants, over half (n = 12) had parents in higher managerial professions, with the pupils from the independent schools having parents exclusively from this socioeconomic group. However, the range of parental occupations of the remaining focus groups was broad.

The findings are presented below, starting with features of a career in dentistry, followed by consideration of the influences on these perceptions. The themes are illustrated by quotations, labelled to provide insight to participant and focus group number, the degree which the participant identified they were hoping to study at university (d = dentistry, m = medicine, o = other or u = undecided), sex, ethnicity, school type, and school stage at the time of data collection (A = A-level of G = GCSE). As an example, the following identifier would be participant 6 from focus group 14, who was hoping to study medicine or dentistry, female, White, from a lower-performing state school, and studying A-levels at time of the data collection: P6,14,m/d,♀,W,↓s,A.

### Features of a career in dentistry

The perceptions of dentistry were considered in relation to sociodemography and school type and stage.

The following key themes emerged from the analysis and will be presented in the following way:

- Attractive features of the career - 'pull factors'
- Unattractive features of the career - 'push factors'.

### Pull factors

Many characteristics of a career in dentistry were identified by the pupils as those that would motivate them personally towards this career. The main dimensions (an adapted model developed by Crossley *et al.*, 2002) are identified below.

#### Science-based career

Perhaps unsurprisingly given the academic level of the pupils and that the sample participants were 'science-minded', dentistry being a science-based career was the most commonly identified factor among the participants as to the appeal of choosing dentistry. With the exception of one student, discussed below, all pupils identified dentistry's scientific nature as a dominant factor. The appeal of a science-based degree revealed four separate motivating concepts. First, that the pupils enjoyed studying science subjects; second, that the pupils were achieving academic success in science subjects; and third, the perceived importance of scientific knowledge within the wider world. These concepts are demonstrated in the following quotes:

- *'Well I kind of have a passion for like science and in general science and maths are like my best subjects and then I think I take to the contents better than most subjects'* P1,3,m/d,♀,B,↓s,A
- *'Science is the foundation of everything'*. P5,3,d/m/o,♀,OA,↓s,A.

The fourth concept was that dentistry as a career offers the opportunity to apply science which became evident within the focus groups and was a commonly cited factor, both directly and also with the identification more specifically of an interest in human anatomy, as demonstrated by the following quotes:

- *'It's the application of science'* P4,7,d, ♂,OA,↑g,A
- *'I've always had like a deep interest in how the human body functions because it is actually quite amazing and something that not a human can come up with so I want to actually kind of live my life with a lot of science knowledge'* P6,3,d/m,♀,In,↓s,A.

The identification of this feature of the profession being a motivating factor was universal, regardless of age, sex, ethnicity, school type and socioeconomic group.

#### Social status and security - extrinsic rewards

Within this dimension, three themes were apparent in the attractive characteristics of dentistry. The first extrinsic reward was income and was identified in all the focus groups. The importance of this factor varied considerably among the d-pupils. For some of the pupils, this was identified as the crucial attraction (and for the pupil below, the only attraction):

- *'I'm really really really money orientated. I'm not going to lie [...] and I kind of researched it and I was, like, oh yeah, this has good pay [...] yeah pay is a big factor'* P2,8,d,♀,In,↓g,A.

But to others, it was a secondary consideration:

- *'I don't think anyone's mentioned it yet but the salary is quite good as well'* P4,3,d/m,♀,OA,↓s,A.

However, although the level of income dentists can achieve was identified by some of the d-pupils as an attractive factor of the career, its relative importance in their career decision-making process was reduced, or was presented in a more covert manner:

- *'I mean, obviously a lot of people have the conception that they are rich. But um, that's not the reason why I wanted to do it'* P3,5,d,♀,OA,↑i,A
- *'[The money] is a bonus really'* P3,8,d,♀,OA,↓g,A.

The female pupils and those of Asian ethnicity identified income far more readily as a motivating feature of the profession than their male counterparts and was the only feature of the career that participants from the academy school could perceive as a motivating feature.

Second, the high status of dentistry as a profession was a readily presented motivating factor:

- *'I think as an adult you are very aware that it is a respected profession'* P7,4,d,♀,I,↑i,A.

This was readily linked to dentistry as public service, relevant to society, with the responsibility of this role requiring trust:

- *'I think those two university degrees [medicine and dentistry] really help the public'* P1,3,d/m,♀,B,↓s,A
- *'It's also being an integral cog in a community, you are portraying a really pivotal role'* P1,8,m,♀,In,↓g,A
- *'You don't believe that he (the dentist) would do anything wrong to you. You have to put a lot of trust in the dentist'* P3,7,m,♂,OA,↑g,A
- *'But actually, if you think about how important they are, because every single person has a dentist'* P4,6,o,♀,W,↓i,A.

As demonstrated in the quotes above, this was important to both male and female pupils from all school types, ethnic groups and socioeconomic status. However, dentistry was perceived as providing a health service with lower levels of responsibility than medicine, which was comparably a motivating factor of responsibility-averse informants from the academy school, as demonstrated in the following quotes:

- *'[After another participant asked if it is better being a doctor or a dentist] dentist - because you don't really risk someone's life you just risk their teeth*' P3,10,u,♀,W,↑a,A
- *'I just wouldn't want their health to depend on me'* P1,10,u, ♂,W,↑a,A.

This comparative motivating feature was identified in other focus groups, by both male and female participants, as demonstrated in the following quotation by a male state school pupil:

- '*If I was a doctor, I'd feel too pressured, 'cos there's quite a lot riding on it if you were like a surgeon or something, so I though dentistry would be best'* P2,1,d,♂,W,↑s,A.

It must be noted that this identification of a lower level of responsibility as a motivating feature of the career was expressed by pupils from lower socioeconomic groups.

Finally, job availability and job security were dominant motivating factors identified by the pupils towards dentistry, perceiving that, after studying dentistry, there would be guaranteed employment and a 'job for life', as shown in the following quotes from female pupils of differing ethnicities and school types, yet all from high socioeconomic groups:

- *'I know that the job guarantee is going down. Because I know it used to be like 100% or 99% and now it's like, by the time we finish, we won't be guaranteed a job as much anymore. (but it will be easy) because you have got a qualification that is needed. There will always be a demand*' P3,5,d, ♀,OA,↑i,A
- *'The idea that it is a career for life. Like, we will always need dentists [...] and [there is] quite good employment, you know, job prospects or whatever'* P2,8,d,♀,In,↓g,A
- *'It's just a stable profession'* P1,3,d/m,♀,B,↓s,A.

#### Structure of service provision

Within this dimension, three themes were revealed as characteristics that would motivate the pupils towards dentistry - 'business', 'structure' and 'self-governance'. First, that a career in dentistry would provide the ability for the pupils to be involved in a healthcare business, as demonstrated in the following quotations:

- *'I just think dentistry is a [...], you know, it's healthcare business'* P7,4,d,♀,In,↑i,A
- *'I'm not sure if it's [business] something I'd necessarily do, but I like to have that option'* P6,5,d,♀ In,↑i,A.

This career feature was introduced to the discussions solely by the Asian participants from the higher achieving independent and grammar schools, of both sexes, involved in the study. Although it was a motivating factor for these participants, for the female pupils from the lower achieving grammar school, it was a demotivating feature, involving two concepts - the individual's perceived lack of aptitude for business and the resultant pressures from running a business as well as practising dentistry, as demonstrated in the quotes below:

- *'I don't have a really, business mind or anything so that's not really something I'm driven towards*' P6,8,d,♀,In,↓g,A
- *'I think it's [the business side of dentistry] a lot of stress on your shoulders as well'* P3,8,d,♀,OA,↓g,A.

The balance between a working life and leisure was a readily identified attractive feature in dentistry, such as flexible, regular (9-5) working hours, as demonstrated in the following quotes from female participants from a higher-achieving independent school:

- *'I think they have quite a good lifestyle. You come home, it's not like working 'til late which you know a lot of people have to do'* P6,5,d,♀,In,↑i,A
- *'You set the hours, you do as much or as little as you like'* P2,5,m,♀,B,↑i,A.

Within this feature (the flexible yet regular working hours), the resultant ability to support family life was crucial to the pupils. This was identified by both female and male participants, from all school types and ethnicities, as the following quotes demonstrate:

- *'You've got to think about when you are older and you've got a family and you want to spend more time with your family. With dentistry you've got more time to be with your family and weekends to enjoy yourself'* P4,7,d, ♂,OA,↑g,A
- *'You can, um, have a kind of family life as well as a work life*' P2,5,m,♀,B,↑i,A
- *'He [cousin] has got a family so he preferred orthodontistry because he works 9-5'.* P5,3,d,♀,OA,↓s,A.

This feature was raised by male and female pupils as a motivating feature, suggesting that traditional roles of men and women in relation to career requirements may not be as apparent among some of the participants as they may have been for previous generations. However, they still persist for others, as shown in the quote below:

- *'Dentistry, I think it is just more flexible and especially [when] trying to plan a family afterwards for women, that kind of thing, but yeah'* P3,5,d,♀,OA,↑i,A.

The importance of this feature of dentistry was most notably revealed when comparisons between the professions of dentistry and medicine were made, with the dimension of service provision being the most commonly identified motivating factor towards dentistry compared to medicine. From the discourse among the young people, the clarity of the comparative decision-making process towards dentistry in relation to medicine was affirmed, especially for female participants:

- *'I think that more people are applying because they are realising that maybe it's probably a better career at the end, like the lifestyle and everything. It's probably why people choose it'* P7,4,d,♀,In,↑i,A.

Not only did a structured working day motivate pupils towards dentistry, pupils identified the profession providing structure within the working day, which appealed to them - the timed appointment slots with designated treatment:

- *'I really enjoyed the working day. I just thought it was structured'* P7,3,d,♀,OA,↓s,A.

Although being part of a team (as discussed below) was perceived as a key feature of the profession, the fact that dentistry could allow for 'self-governance' was also integral:

- *'You get your independence to do your work. And I think, you know, you can actually do your job. And I think that's what I like about it'* P7,4,d,♀,In,↑i,A.

Two further concepts within the dimension of the structure of service provision were exposed during the comparative discourse with medicine - 'the working environment' and 'whole case treatment'. The working environment was identified as a demotivating feature by pupils potentially applying to study medicine, but a motivating feature for the prospective dental applicants, as demonstrated in the quotations below:

- *'As you walk in there it's got a bit of a different feel. It's relaxed, it's not so hectic'* P2,2,m,♂,W,↑s,A
- *'It's not as rushed as doctors and stuff in hospitals [...] more fun, more relaxed*' P2,9,d, ♀,OA,↓f,A.

Medical and other pupils also perceived 'whole case treatment' as an attractive feature of the job, albeit that they did not propose to study dentistry and was a concept that was not identified directly by dental pupils within the focus groups. This motivating feature links not only with the nature of service provision within dentistry, but also with the dimensions of social interactions and the status of the profession. These additional concepts again reveal the importance of comparisons with medicine within the decision-making process for prospective dental pupils.

#### Career opportunities

The specialised nature of dentistry was a motivating feature for informants who hoped to study dentistry as a degree. Many of the prospective dental pupils identified that dentistry is already a 'specialism within medicine' and this attracted them towards the career but was complimented by the recognition of the further career opportunities within the field, with technological advances necessitating the need for career-long learning:

- *'Dentistry is obviously a lot smaller, narrower, so I just thought it must be more fascinating, more detailed, if it's five years that's just the mouth*' P3,5,d,♀,OA,↑i,A
- *'I think it's just because dentistry is always changing and, er, constantly learning something new throughout your career'* P6,8,d,♀,In,↓g,A.

Within this perceived specialism was the appreciation of potential career diversity, not only within the working day, but also future career opportunities. This was recognised by pupils from all school types and ethnic groups:

- *'You don't have to work in just a general practice, you can work in a hospital or in like the community and that kind of thing, so there are more options than other people think, it's not just working in dental practice'* P6,5,d,♀,In,↑i,A
- *'Well I think it's quite exciting like anything could happen, for example with dentistry you could go in to work with days going like sort of normalish if you can really say normal and then you might have like an emergency patient, I don't know, it just sounds so exciting like*' P1,3,d/m,♀,B,↓s,A.

Interestingly, the diversity of the career was linked with status, as demonstrated in the following quotation from an Indian female pupil at a lower-performing grammar school:

- *'And one thing that really interested me [...] dentists actually detect before doctors do. That's so cool. It's not just like, ulcers and stuff'* P4,8,d,♀,In,↓g,A.

#### Social interactions

The perception that dentistry is a profession which allows for interaction with people was identified as a factor that made it attractive as a potential future career. Within this theme, three main concepts seem to appeal to the pupils.

First was general social interaction:

- *'Socialising with a variety of people'* P2,3,d,♀,B,↓s,A.

Secondly was building and sustaining relationships with patients. Although there was recognition that patient management may be demanding, this could only add to the diversity of the working day:

- *'I think that the dentist focuses more on the care side, you can sit with your patients a bit longer, you can, erm, you know, really build a relationship with them'* P7,4,d,♀,In,↑i,A
- *'During my work experience, my dentist, he saw a patient and he was like, "yeah, she's been seeing me for about you know, 30-40 years"'* P6,8,d,♀,In,↓g,A
- *'You probably will have difficult patients every day but I think that's part of the challenge'* P7,4,d,♀,In,↑i,A.

Again, the importance of this feature of the career was presented comparatively with medicine, as shown in the following quote from a female pupil from a lower-performing grammar school:

- *'When you are a dentist you are there from start to finish and you get to know your patient well, whereas with medicine...'* P5,8,m,♀,OA,↓g,A.

Interestingly, the interaction with patients and the skills required for the role was linked with the dimension of social status (public service being 'irreplaceable') and job security:

- *'The social aspect of the dentist, trying to calm you down or something, that will never be, that's irreplaceable, yeah. That's why dentists will always be needed so it's a really secure job'* P1,7,o,♂,In,↑g,A.

The third concept was the identification of the interactions as part of a team:

- *'That team ethic and that teamwork and that strong friendship between your team. And, erm, I think the dentist is usually at the top of it. So, it's the teamwork and the socialising as well [...] I saw like, [dentistry] was one of the very few professions that actually as a dentist you are a team leader'* P3,8,d,♀,OA,↓g,A.

Within this concept, it was not only being part of a team that was important to the pupils, it was being the leader of a team - this links with the dimension of professional status, not just perceived within society, but also the higher status among professional colleagues.

'Interaction with people' was not only perceived as a key motivating characteristic towards dentistry, but was also a factor in active rejection of other career choices:

- *'I just decided, because with maths you can't, you don't work with patients, customers and people as much and I think that's what I wanted to go towards, working with people'* P3,5,d,♀,OA,↑i,A
- *'You get to create a relationship with the patients, rather than in business you're just on a computer or something, kind of boring*' P1,9,o,♀,OA,↓f,A.

Dentistry was identified, therefore, as a career which would inhibit social isolation and this characteristic was far more apparent among female participants than male participants.

#### Personal skills and care - intrinsic rewards

The concept of helping people was an important motivating factor towards dentistry for many of the pupils who identified their caring nature being fulfilled by a career in dentistry.

Consideration of a science-based career that allows for the provision of care appeared to have been central to d-pupils' choice of dentistry. This is demonstrated in the following quote by one student in response to the question 'what is most important when choosing what to study?':

- *'Subjects you've found interesting and the fact that I wanted to help people are the main thing'* P2,1,d,♂,W,↑s,A.

Within the concept of 'helping people' was the identification that the provision of this service would have impact on their patients' lives:

- *'[Oral health] affects someone's life so much they don't realise it until they actually have bad teeth. But I do think it does affect, you know, self-esteem, um and your whole, your whole confidence I think it is affected by your teeth'* P7,4,d,♀,In,↑i,A.

This quote illustrates how the participants linked 'helping people' with the 'status' of the profession, as through their care, they can impact on the mental wellbeing of their patients.

Other intrinsic rewards from this profession were the ability to utilise personal skills for professional fulfilment, most notably manual dexterity:

- *'It's the application of science with technical manual work'* P4,7,d,♂,OA,↑g,A.

As well as with technical skill:

- *'And there's a lot of machinery to work with as well. Like the vacuum and the drill and everything'* P4,3,d/m,♀,OA,↓s,A.

Plus, the opportunity to employ artistic expression:

- *'[You're] also an artist and you know you use your mind in very creative ways from a day to day basis and that really appealed to me. I love being artistic and using my hands and in very intricate ways so that really appealed to me'* P3,8,d,♀,OA,↓g,A
- *'[Dentistry compared with medicine] would be better because I've got good manual dexterity because I do art and stuff'* P1,7,o,♂, In,↑g,A.

The importance of intrinsic rewards from the practising of dentistry was identified by both sexes and different school types. However, for one potential medical student from a lower-achieving independent school, it was this feature of the career which allowed them to discount dentistry as a potential career option:

- *'I'd prefer not to do that fully hands on thing. I'd rather just be there and talk to them'* P2,6,m,♀,W,↓i,A.

Interestingly, one further personal benefit, specifically from dentistry, could be identified:

- *'You can make sure your kids have perfect teeth*' P3,1,m,♂,B,↑s,A.

This quotation from a Black, male student from a high-performing state school links an intrinsic reward of dentistry with the nature of service provision, in that it can benefit a family life.

#### Vocational degree

In addition to the above themes, an auxiliary theme emerged, which related to the attraction to studying dentistry (as opposed to dentistry as a career). This factor influenced the pupils who wished to learn a vocation at university so that they would be readily employable once the degree was completed:

- *'To be a dentist you can just go straight in to a practice, I just think you're learning a skill, not a subject, if that kind of makes sense*' P1,3,d/m,♀,B,↓s,A
- *'To find not just a degree but a career [...] I think personally then a profession is suited to me because I like knowing where I'm going'* P7,3,d,♀,OA,↓s,A.

It was also identified that an advantage of studying dentistry at university was that it would be a national vocational degree - the Bachelor of Dental Surgery qualification is not graded (like other degrees, for example, First, 2.1, 2.2, 3), as shown in the quotation below:

- *'[With dentistry], you come out with the same degree wherever you go to [university]'* P1,5,m,♀,M,↑i,A.

### Push factors

Three demotivating features of a career in dentistry were identified by the pupils, which are explained below.

#### Lack of diversity

Two prominent areas of lack of diversity within the career were identified - the working environment and the service provided:

- *'They need to liven up the work environment a bit. Which could be why they're suicidal. If my everyday life was like that I'd probably at some point rather get really annoyed with it* P1,2,m,♂,OA,↑s,A
- *'I think one of my reasons also was 'cos I think, no offence to dentistry, but erm, like, if you looked at the mouth all day you'd be a bit bored. But if you like, if you are a doctor, you look at every little thing in the body and you kind of have new experiences everyday*' P1,4,m,♀, OA,↑g,A.

#### Societal perceptions and low status

The concept that dentists are thought of negatively in society was a clear demotivating factor associated with dentistry. After being asked what would be negative about being a dentist, some replied:

- *'The social perception*' P7,7,m,♂, OA,↑g,A
- *'People generally hate you. Or dislike you, hate is a strong word'* P3,1,m,♀,B,↑s,,A.

Although the perceived high social status of dentistry was a motivating feature of the career, the perceived higher comparative status of medicine was a clear motivating factor for the pupils wishing to study medicine, regardless of school type or ethnic group:

- *'Also some people might do it [medicine] for the prestige and for the name so, I don't know, maybe doctors are looked at better than dentists so that's why people want to be doctors and not dentists'* P4,3,m/d,♀,OA,↓s,hSEC,A.

Although all of the pupils perceived that dentistry has a lower social status comparatively to medicine, the level of responsibility and resultant stress was identified by some of the prospective dental pupils as a negative feature of the career:

- *'It's high stress. There's pressure from every side*' P7,3,d,♀,OA,↓s,A
- *'In many cases the dentist is the boss and the leader and you have no-one to look to for advice, you have to make the decisions. And I think that's, I think that's probably where the suicide rate, drugs and alcohol comes from!*' P3,8,d,♀,OA,↓g,A.

#### Anatomical region

A specific feature of dentistry that was a universally-identified push factor away from the career was an aversion to working with teeth, the mouth and the required proximity to the patients:

- *'I thought about it [dentistry]. I just don't like poking around in someone's mouth.' ("But you poke around in people in medicine?") 'But they're not looking at you while you are doing it'* P1,1,m,♂,W,↑s,A.

### Influencers on the decision-making process

As well as individual perceptions of dentistry as a potential career and their influence on young people when considering their university choices in relation to age, sex, ethnicity, socioeconomic status, the influence of environmental context was revealed, which the pupils themselves recognised:

- *'The media, we hear opinions from different people, friends, family and on the internet and that might also shape your views of what you want to go in to'* P5,3,m/d/o,♀,OA,↓s,A.

**Fig. 1:**
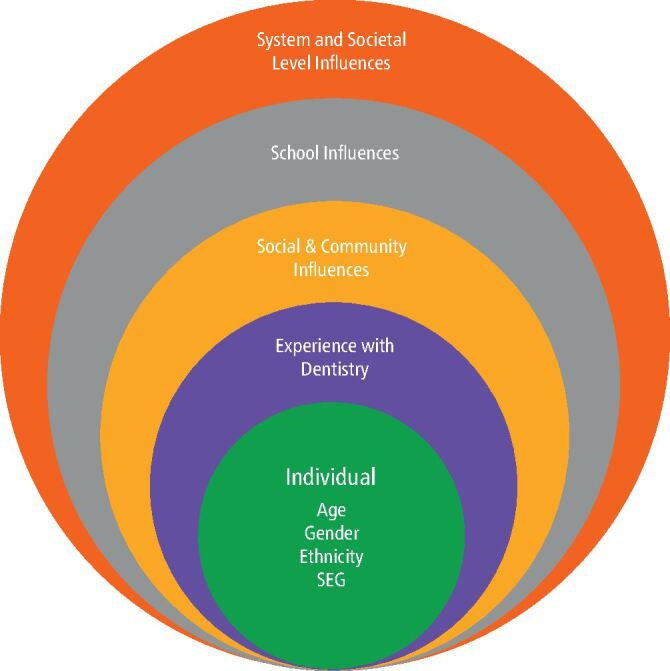
Individual and environmental influencers on the perceptions of London secondary school pupils on dentistry as a career

These individual and wider influences on career decision making are shown in Figure 1.

### Experience with dentistry

For nearly all of the d-pupils, their personal experience at their dentist was positive and the importance of this was identified by one student:

- *'Growing up I always had a really positive view of dentists because it starts when you're young and you go to the dentist and [get] positive feedback*' P7,3,d,♀,OA,↓s,A.

Many identified positive personal experiences at the dentist as an initiating influence towards this career, but negative experiences were conversely a demotivating factor:

- *'I got into dentistry because I love how my dentist is in to contact with people and she treats her patients really well, including me'* P2,3,d,♀,B,↓s,A
- *'They're scary. I did [think about doing it] for a bit then I got scared of my dentist'* P8,9,o,♀,B,↓f,A.

These experiences were most related to orthodontic treatments, which occurred in early adolescence at the age of 13 or 14 years when GCSE decisions are occurring - the first time young people narrow their educational choices, with work experience being an initiating or reinforcing influence:

- *'The first time I was really interested going in to dentistry was after completing my two weeks work experience in Year 10*' P2,3,d,♀,B,↓s,A.

### Social networks

Family members and in particular, parents, were the dominant source of influence on the pupils when deciding to study dentistry at university. This ranged from parents facilitating and encouraging pupils to explore a range of career options and providing the initial suggestion of a career in dentistry, to actively promoting dentistry itself. The reasons for parents suggesting and encouraging pupils towards a career in dentistry were identified as the perceived status of the profession, the ability to support family life and job security:

- *'My mum always flags this up, but when you're a girl and you have kids, things like that, she says, "think about family life because obviously you're not a man, you're not going to be out there all day long"'* P3,8,d,♀,OA,↓g,A
- '*Yes, my parents are happy with dentistry. As long as you're called a doctor'* P2,9,d,♂,OA,↓f,A
- *'He will be [proud of me], because I think my dad always wanted me to do something where I* *had a stable job afterwards*' P6,5,d,♀,In,↑i,A.

These family perceptions were most readily identified among pupils from Asian ethnic groups, regardless of school type, sex or socioeconomic status, highlighting the importance of cultural expectations:

- *'I wouldn't have wanted to do something they [my parents] didn't feel was right for me or anything'* P4,7,d,♂,OA,↑g,A.

Pupils who had families employed in healthcare professions identified exposure to healthcare culture and were also influenced by parental 'expectation' and the promotion of healthcare professions as an alternative to their/sibling's careers:

- *'I've kind of grown up around a medical background um and all our family friends and stuff are in that kind of sector so'* P6,8,d,♀,In,↓g,A.

Conversely, the informants who did not come from a family employed in healthcare identified did not identify any such expectation:

- *'My parents come from a non-sciencey background. So they didn't really know much about medicine or dentistry so they didn't steer me in any way but they were happy with whatever choice I made really*' P2,3,d,♀,B,↓g,A.

A key feature of parental influence was many of the d-pupils' parents initially expected or suggested that the pupils study medicine and the m-pupils cited this expectation also, due to the higher status of medicine, cultural influences and the converse presence or lack of other doctors within the family. This was especially notable among the pupils from minority ethnic backgrounds, of both sexes and from all school types, where parental influence was definitive:

- '*My parents work in ITU [...] I was always told when I was young like "you're going to be a doctor". So yeah*' P6,1,m,♂,B,↑s,A
- *'My mum [made me decide to do medicine]. She wants to become the mother of a doctor'* P3,1,m,♂,B,↑s,A.

As well as parents, wider family members played an influencing role on the pupils' decision making, particularly siblings, if they were involved/linked to healthcare professions:

- *'Then I just didn't want to [to do medicine], because seeing the stress that they went through I thought, I don't know, I just thought I wouldn't be able to cope as well as they [my brothers] would maybe with medicine'* P3,5,d,♀,OA,↑i,A.

The opinions of 'wider family' was particularly noted among Asian pupils, especially in relation to the cultural perception of the greater status of doctors over dentists. For many of the prospective dental pupils, most notably those from lower socioeconomic groups, minority ethnic groups and from families who were not employed in health services, the justification of pursuing dentistry instead of medicine was revealed, after being asked 'what do your family say when you say you are wanting to study dentistry?':

- *'Nothing. No, because I first said medicine and they seemed excited about it, but when I said dentistry they thought it's not as higher status as medicine so it's sort of downgraded a little bit. But after I explained to them that it's as hard as med school then they understood'* P2,9,d,♂,OA,↓f,A.

### Friends

The reactions the d-pupils had encountered when informing their friends that they were considering dentistry was a key influencer in the decision-making process. These attitudes varied between pupils, but not among school types. Some attitudes were negative, for example:

- *'Yeah I think [dentistry has a negative image at this age]. Because when you are at school if you say "I'm doing medicine" everyone says "wow!", but if you say "I'm doing dentistry" [they say] "why would you want to stare in someone's mouth all day?"'* P6,5,d,♀,In,↑i,A.

Or these attitudes were a source of active support, which is most notable for pupils considering a career in dentistry where other social determinants of support may have been lacking - in the lower performing state school where information provision was minimal, or pupils from lower socioeconomic groups or whose parents were not in healthcare professions:

- *'So my friends always encourage me and they say that yeah it's something good, it's ambitious'* P7,3,d,♀,OA,↓s,A.

Many sources of professional advice were family members and family friends who provided positive reinforcement in the dentistry decision-making process, which was amplified for the pupils whose parents were from lower socioeconomic groups in non-healthcare employment:

- *'We're quite close with our dentist and she's a family friend as well so, yeah, they [my parents] see her doing a good job so they're happy with it'* P2,3,d,♀,B,↓s,A.

Pupils that did not have dentists or dental pupils as part of their social network recognised this as a disadvantage and discussed it with a negative emphasis, as demonstrated in the following quotation:

- *'I have no one in the family who is a dentist, they are mostly medics. Yeah, I haven't really heard that much about dentistry from the side of students or anything'* P3,5,d,♀,OA,↑i,A.

### Community

As well as family and friends, the wider social community was identified as a source of influence on the pupils' perceptions of dentistry within their career decision making by those from minority ethnic groups:

- *'Well honestly they say [in my community] that dentists are thieves because they take their money and every time they go there they end up spending more than they wanted and more than they think they needed and so they almost all have that negative view and they told me not to do it'* P3,3,d,♀,OA,↓s,A.

The broader environmental influence of a pupil's community was an amalgamation of an individual's ethnic, social, societal and cultural influences, with the synergy between family expectations, social status of the career and community cultural ethos being directly expressed by students from minority groups:

- *'The status thing, being a doctor kind of thing, I think they think the next best thing to a doctor is a dentist [...] I think it is the Asian thing! No, but I don't know what it is, but they did push me very very far. [Dentistry] is still a doctor. If I ever said I wanted to be a doctor* they would *jump for joy. But no, I once said it and it was a joke and my dad got really annoyed and was like "you shouldn't joke about things like that!"'* P2,8,d,♀,In,↓g,A
- *'Well, my parents basically told me they'd support me whatever I did but my grandma, who was Jewish, was very much, well she wants me to go to medical school [...] to have a stable career [...] and bragging rights, that's all it is, yeah! Bragging rights'* P2,5,m,♀,B,↑i,A.

For pupils from high socioeconomic backgrounds, being raised in a familial culture of higher education and a professional career directly lead to these expectations upon the participants - a shared pro-educational ethos, or 'family career pathway' - as demonstrated in the following quotation by Black female student from a higher attaining state school:

- *'My background is that everyone's quite educated [...] I'm not going to withhold it, they [parents] have a different ethnic background, they have higher expectations, because they worked to get where they are, so they want you to exceed that place. Because for English people, white people, they got to their position a lot easier, so for a Black person to get to the stage of a doctor, it's a really big achievement, or an Asian person'* P7,2,m,♀,B,↑s,A.

### School

Three sources of influence on the perception of dentistry as a career came from the school environment, which varied considerably between the different school types. First, their career teachers' opinions; second, the source of advice provision from careers departments; and third, their network of peers who are interested in similar careers who provide a source of information:

- *'They [teachers] go on about how competitive it is and how hard it is to get into in the first place*' P7,9,m, ♂,OA,↓f,A
- *'You know normally we have outside speakers who come in and talk to us? We never had a dentist'* P3,5,d,♀,OA,↑i,A
- *'It's not, you know, you are not made as aware of it as other careers, so you have to kind of find it yourself, which I think wasn't good'* P7,4,d,♀,In,↑i,A.

### System and societal level influences

#### Media perceptions

There was universal acknowledgement among the pupils that dentistry is portrayed negatively in the media (news stories, TV and movies) and that this was perceived as unjust, especially compared with the positive portrayal of a career in medicine. It was a recognised negative factor when the pupils were considering a potential career in dentistry as it reduced the social status of the career:

- *'Yeah, it mostly has an influence on most people's perception of anything really [...] because people don't really like dentists, they think they are evil, they think "my dentist automatically by default is also evil", though it's not necessarily true. It's the problem with people's perceptions. But it can hinder people's, like, choice because they wouldn't get the respect they would if they did something else'* P3,7,m,♂,OA,↑g,A
- *'It was probably two or three years ago [that I decided I wanted to study medicine] when I started watching Grey's Anatomy and watch all the idealistic doctor's lives and all that'* P4,13,m,♂,In,g,G.

#### Public perceptions

The public perception of dentistry in the UK was explored both generally (in society as a whole) and also generationally. All the pupils agreed that the public perception of dentistry was very negative, based on a culture of fear and features of the career:

- *'You tell someone you're a dentist then a lot of people feel wary or intimidated that you're a dentist and if you're a doctor then doctors are always perceived in a good light because they help people*' P7,3,d,♀,OA,↓s,A
- *'I hate it because, whenever I tell someone I want to be a dentist, they're, like, "yeah, they have a high suicide rate"'* P2,8,d,♀,In,↓g,A
- *'They just think I'm after the money. I just think it's an unfair, kind of...'* P6,8,d,♀,In,↓g,A.

#### Economy

As explored above, a dominant motivating factor towards studying dentistry at university was that it was a vocational degree, which resulted in a 'stable, professional' job with a high level of income. At the time of data collection for this study, the UK was in economic recession and this, coupled with the introduction of higher tuition fees at university, was cited by the pupils as an influence towards this career:

- *'It's a more stable profession to have, in this climate, people realise it is better to have a stable job with a stable income'* P3,8,d,♀,OA,↓g,A
- *'[Medicine] leads to a stable career at the end. And with the recession and everything it seemed like a good place to go towards right now'* P3,7,m,♂,OA,↑g,A.

#### Policy

Although it was not cited during the focus group data collection, two pupils at the end of the session asked 'off the record' whether I knew of any current admissions policy of 'positive discrimination' to UK dental schools. These pupils were from an independent school and were concerned that they would be discriminated against in this climate of widening participation when applying to dental school, in favour of pupils from less privileged educational backgrounds due to the implementation of quotas. These pupils were considering medicine as a career also and were not concerned about such a policy when applying to medical school. This perceived educational admissions policy may have acted as a potential demotivating factor when these pupils were applying to university but this is conjecture, given the anxiety these pupils demonstrated when asking about UK dental school admissions policies.

## Discussion

### Summary and importance of findings

Seven dimensions of motivating features of a studying for a career in dentistry were identified by London secondary school pupils and three demotivating features - push and pull factors. The importance of these features were relative to individual sociodemographic characteristics and the wider environmental context. It is crucial that we listen to the views of young people about a career in dentistry earlier in their career decision-making journey, before they have committed to a career pathway, to deepen our understanding of the motivations of the future workforce and inform professional and policy decisions.

### Strengths and limitations

By focusing on science-minded GCSE and A-level pupils for this area of the research, it must be acknowledged that this presupposes pupils who have already chosen to study science. The bias of this sample can be justified as these pupils were chosen as they had already started to meet, or partially meet, the stringent entrance criteria necessary to study dentistry. Not all secondary school pupils will have the academic ability nor the interest in studying science, which are essential prerequisites of any student hoping to study at dental school. Although the sample population for this research was from London only, further comparative research could be conducted in other regions of the UK, urban and rural, particularly among underrepresented groups in dentistry. The data were collected in the years pre-COVID (2011-2017), the pandemic having a major impact on admissions processes and clinical care. Nonetheless, many of these issues remain, with new challenges being presented during lockdown and with national social and educational limitations emerging.

### Motivation towards dentistry

The motivating features towards a career in dentistry identified in this research closely relate to the dimensions suggested by Crossley and Mubarik and other studies, when investigating the influences on undergraduates studying dentistry and medicine.^6^^,^^14^^,^^15^^,^^23^^,^^24^^,^^26^^,^^34^^,^^35^^,^^36^^,^^37^^,^^38^ However, through exploring the perceptions of the students at an earlier stage of their academic journey involved in this study, seven pull factors were identified: 1) science-based; 2) status and security - extrinsic rewards; 3) structure of service provision; 4) career opportunities; 5) social interactions; 6) personal skills and care - intrinsic rewards; and 7) a vocational degree.

As dental students are the future workforce, it is important to understand the motivators towards this career^6^ but this had yet to be explored in pupils who were yet to make their career choice. This may be particularly relevant given that dental school applicants may finalise their career decision within one year of university application.^39^ Although the pull factors pupils report towards a career in dentistry support those identified by students already embarking on a dental degree, the pull factors had yet to be explored. Studies investigating the perceptions of secondary school pupils of career in medicine similarly report the importance of environmental context, rather than low levels of factual knowledge, on the development of medical ambition and career identity in young people from disadvantaged backgrounds.^28^^,^^30^

The importance of a science-based career was not unexpected given the educational level of the participants and they were invited to participate based on a proven academic ability. The extrinsic rewards of dentistry were universally identified - the perceived high professional status of the career was identified as a key motivating feature by all the participants, as was income, which was most readily expressed by Asian and female informants. Dentistry as a 'healthcare business' was a motivating feature for Asian participants. While flexible, regular working hours allowing for family and work-life balance were identified as an attractive feature of the career by pupils of both sexes, stereotypically traditional female roles were still apparent. Job security, an extrinsic reward, was universally identified by pupils from higher socioeconomic groups. Career diversity, social interactions, intrinsic rewards and dentistry as a vocational degree were important to all pupils, regardless of age, sex, school type, ethnicity and socioeconomic classification. These pull factors of a dental career may in part explain the proportion of female and Asian pupils applying to study dentistry.^10^^,^^13^ The perceived push features of dentistry motivated pupils towards medicine, or were factors that lead to the early dismissal of dentistry/an explanation of why dentistry had never even been considered. Although previous research has highlighted the importance of family relations who work in medicine or dentistry influencing students towards a career in dentistry, this investigation has revealed that a personal connection with a healthcare professional (at whatever level) within their social network helped affirm the aspirations of pupils ^6^^,^^26^^,^^39^

### Factors pushing pupils away from dentistry

This research, as well as adding to the understanding of features of a dental career which motivate secondary school pupils towards dentistry, reveals the converse push features which may inhibit students from pursing this career. The perceived features of the job which influenced students away from this career had, to date, not been explored and the revealed three dimensions of push features are: 1) lack of diversity; 2) societal perception and low status (relative to medicine); and 3) aversion to the anatomical area. The perception that dentistry requires a lower skill mix compared with medicine was raised by a GCSE level group, which devalues the service provided by dentists as there is 'self-care' alternatives. This perception may be due, in part, to an improvement in public health awareness in patient preventative care and also the resulting improved oral health of young people who, although may visit their general dental practitioner twice a year, are requiring less invasive treatment and so are not exposed to complex treatments for which dentists are trained. Interestingly, several factors were considered conversely as pull and push features by different pupils, which were the 'business' of dentistry, the level of responsibility a dentist has for their patients, dentistry as a 'specialism' and the 'work environment'. This highlights the significance of individual standpoint. Understanding these perceptions of the profession may be an important aspect of aspiration raising in academically able students, supported by the research of Mathers and Parry among prospective medical students, who argued that widening access might be increased in part by 're-orientating working class perceptions of medicine as a profession'.^40^

### Individual perspective under wider influences

This research highlights that individuals must be allowed to consider their own push and pull factors regarding a career in dentistry as they progress through their career decision-making journey. These qualitative findings also provide insight into the wider environmental influences on career decision making and perhaps help to explain the patterns we have observed in the applications and admissions to UK dental schools in recent decades, with those with most social capital being more likely to gain positions in these professions.^10^^,^^11^^,^^12^^,^^13^ Going forward, it is vital we explore how young people from disadvantaged backgrounds who lack such support can take advantage of opportunities that are now becoming available to them.^41^^,^^42^^,^^43^

## Conclusions

Pupils in London secondary schools report similar features of dentistry as being attractive as dental students, as well as its importance as a vocational degree and although dentistry appears to lack status, diversity and profile when compared with medicine, it may be more acceptable in relation to its lifestyle. Individual pupil characteristics and wider environmental influences interplay in early dental career decision making.
